# Molecular insights into uterine fibroids: crosstalk between SHH and Wnt pathways, DNA methylation, and miRNAs regulation

**DOI:** 10.1007/s00438-026-02491-3

**Published:** 2026-08-29

**Authors:** Grazielle Marques Duarte Purcino, Kelly Pedrozo Ferreira, Nilo Bozzini, Edmund Chada Baracat, Katia Candido Carvalho

**Affiliations:** Laboratório de Ginecologia Estrutural e Molecular (LIM 58), Departamento de Obstetrícia e Ginecologia, Hospital das Clínicas da Faculdade de Medicina da Universidade de São Paulo (HCFMUSP), Av Dr Arnaldo 455, sala 4124, Cerqueira Cesar, São Paulo, SP CEP 01246-903 Brazil

**Keywords:** Uterine leiomyomas, Sonic Hedgehog pathway, Gene expression, MicroRNA, DNA methylation

## Abstract

Uterine leiomyomas (ULMs) are the most common benign gynecological tumors affecting women in reproductive age. Symptoms such as abnormal uterine bleeding, pelvic pain, and infertility may occur, leading to a significant decline in patients’ quality of life. In addition, ULMs represent the second leading indication for gynecological surgery in Brazil, imposing a substantial economic burden on the public healthcare system. However, despite their high prevalence and clinical impact, there is still no curative clinical treatment for these tumors, and the molecular mechanisms underlying their development and clinical behavior remain poorly understood. Recent studies have demonstrated the involvement of the Sonic Hedgehog (SHH) signaling pathway in uterine mesenchymal tumors. Therefore, the aim of this study was to investigate the expression profile and regulation of the SHH and Wnt signaling pathways in ULMs, as well as to evaluate their potential role in tumor progression through integrative analyses of promoter methylation, mRNA expression, and microRNA-mediated regulation. Initially, 106 genes related to SHH, Wnt signaling pathways were examined in ULM, compared to normal myometrium (MM) by real-time PCR. Interactions among SHH pathway genes were assessed through multivariate analysis. In addition, methylation profiles of nine key SHH-related genes were evaluated using methylation-specific assays, and the expression of 84 microRNAs (miRNAs) was analyzed in association with differentially expressed genes potentially involved in tumorigenesis. Differential expression analysis identified 23 dysregulated genes, including 13 upregulated and 10 downregulated genes. Multivariate analysis suggested that SHH pathway activation in ULMs occurs independently of SHH–PTCH1 binding and may involve *GLI1*, *CCND1*, and *BCL-2*. Significant DNA methylation alterations were detected in *PTCH1*, *SMO*, *GLI1*, *GLI3*, *GREM1*, and *WNT1*. Furthermore, 16 miRNAs were differentially expressed, eight of which showed significant correlations with their predicted target genes. Collectively, these findings suggest that ULM pathogenesis involves a complex regulatory network integrating SHH and Wnt signaling pathways, epigenetic modifications, and microRNA-mediated post-transcriptional regulation. These results provide novel insights into the molecular mechanisms underlying ULM development and identify candidate regulatory elements that may serve as potential biomarkers or therapeutic targets in future functional studies on uterine fibroids.

**Supplementary Information:**

The online version contains supplementary material available at https://doi.org/10.1007/s00438-026-02491-3.

## Introduction

Uterine leiomyoma (ULM) is the most common benign gynecological tumor affecting women of reproductive age (Drayer and Catherino 2015; Styer and Rueda 2016; Commandeur et al. 2015). The main clinical symptoms of ULM include abnormal uterine bleeding, dysmenorrhea, and pelvic pain. Recurrent abortion and infertility have been associated with this condition (Drayer and Catherino 2015; Styer and Rueda 2016; Yang et al. 2016). Therefore, reduced quality of life and psychosocial stress are important consequences of ULM (Drayer and Catherino 2015; Styer and Rueda 2016).

At present, hysterectomy is the surgical procedure that is performed most frequently for ULMs. Other approaches, such as laparoscopic and robot-assisted hysterectomy, may also be employed, as they are associated with fewer complications and shorter recovery times (Bonafe et al. 2018). However, these procedures may represent a substantial economic burden for patients and society. On the other hand, there is an absence of a specific curative or effective clinical treatment for ULMs.

Several studies have been conducted to identify biomarkers that may be useful in uterine tumors for differential diagnosis, prognosis, or prediction of treatment response. In this sense, the role of multiple molecules potentially involved in ULM development, such as mutations in the *MED12* (Makinen et al. 2011) and *HMGA2* (Velagaleti et al. 2010) genes, has been assessed. Estrogen and progesterone promote ULM growth through deregulation of signaling pathways involved in cell proliferation, apoptosis, and differentiation (Reis et al. 2016). Several growth factors, such as VEGF, EGF, PDGF, IGF, TGF-α, and TGF-β, have also been demonstrated to play a relevant role in ULM growth (Islam et al. 2016). Two essential features of ULM are increased smooth muscle proliferation and excessive deposition of extracellular matrix (Grudzien et al. 2010).

**Fig. 1:**
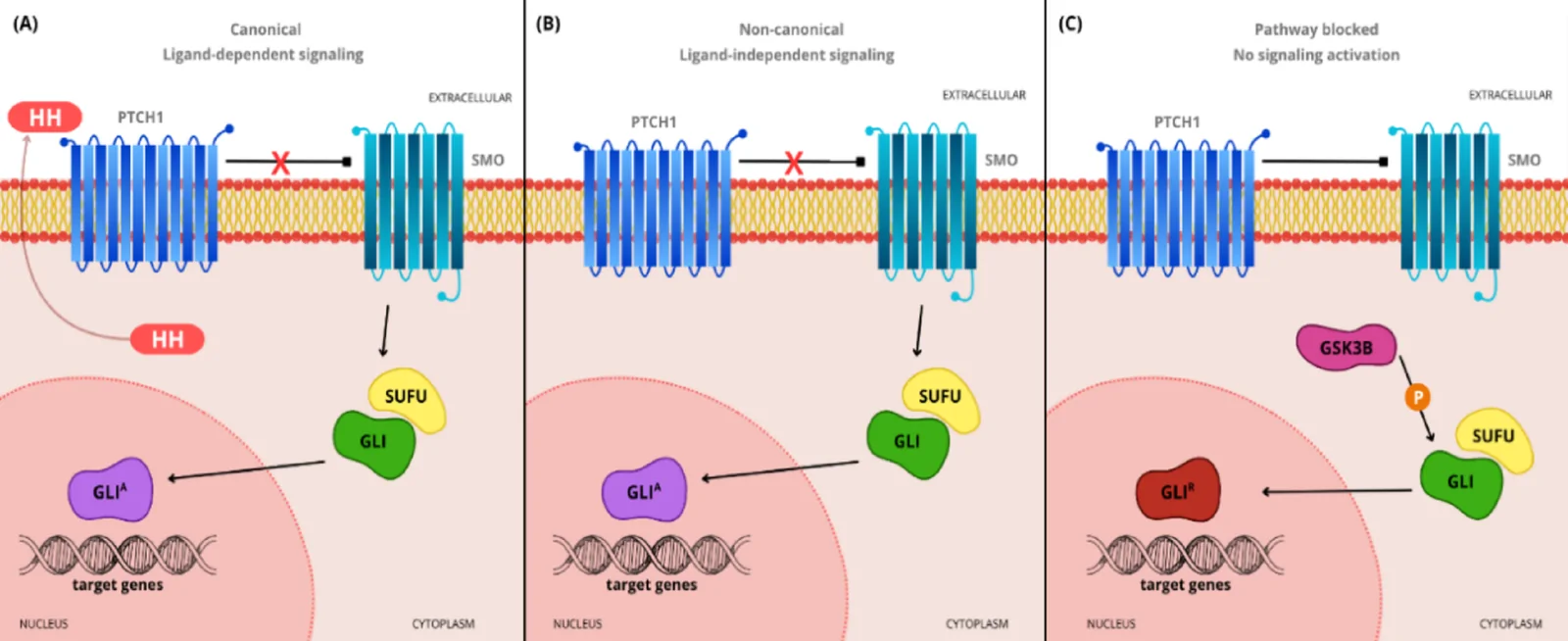
Illustration of Hedgehog (HH) signaling pathways showing the molecular mechanisms of canonical (**A**), non-canonical (**B**), and repressed (**C**) signaling. These pathways involve key regulators such as PTCH1, SMO, GLIs, and SUFU, which were investigated in this study

Genes of the Hedgehog (HH) signaling pathway have been described as playing a role in controlling cell proliferation and progression in a variety of neoplasms (Wicking and McGlinn 2001). As described in the literature, the Sonic Hedgehog (SHH) signaling pathway functions through both canonical ligand-dependent and non-canonical ligand-independent pathways, as illustrated in Fig. 1.

Canonical signaling occurs when SHH ligands bind to PTCH1, which allows SMO activation and subsequent translocation of GLI transcription factors to the nucleus (Garcia et al. 2021a). This process promotes target gene expression. In non-canonical signaling, downstream components, such as SMO and GLI, can be activated independently of ligand binding (Skoda et al. 2018). Inhibition of this pathway is characterized by repression of SMO by PTCH1, which results in the accumulation of GLI proteins in the cytoplasm. This process can occur through the action of SUFU or through phosphorylation of GLI proteins by GSK3B (Garcia et al. 2016). The consequence of these events is the blocking of transcriptional activation.

The SHH signaling pathway plays a critical role in embryonic development, regulating the differentiation and maintenance of tissues (Kotulak-Chrzaszcz et al. 2022). Once activated, the SHH pathway has been identified as a regulator of a large number of cellular processes, including proliferation, apoptosis, cell cycle control, angiogenesis, and adhesion (Garcia et al. 2021b). It has been established that other signaling mechanisms also play essential roles in related processes. Notably, the Wnt/β-catenin pathway has been documented to be overexpressed in leiomyoma cells and has been associated with their formation and proliferation (El Sabeh et al. 2021). The Wnt pathway is a complex regulatory cascade involved in the control of cell proliferation and differentiation. In leiomyomas, its activation has been associated with tumor development and expansion. There have been reports of overexpression of different ligands, such as WNT4 and WNT5A, as well as alterations in inhibitory genes, indicating a significant but still complex role in the pathophysiology of these lesions (El Sabeh et al. 2021).

Another point to consider is the role of epigenetic mechanisms. They have also been implicated in the pathogenesis of uterine fibroids. DNA methylation is an example of regulatory mechanism capable of modulating gene expression without altering the DNA sequence. Previous studies have shown that aberrant methylation patterns may contribute to altered gene expression in uterine fibroids, affecting pathways involved in cell proliferation, differentiation, and extracellular matrix remodeling (Navarro et al.^2012^). Therefore, the evaluation of methylation patterns in selected genes involved in the SHH and WNT signaling pathways may contribute to additional insights related to these tumors.

We previously described alterations in the Sonic Hedgehog (SHH) signaling pathway in mesenchymal uterine tumors (Garcia et al. 2016). In that study, SMO and GLI1 proteins were found to be overexpressed in ULM tissues compared with myometrium (MM), suggesting differential activation of the SHH pathway in these tumors and a potential role in malignant risk. However, the same analysis did not reveal significant differences in the expression levels of the Hedgehog ligand protein (HH), the main activator of the canonical SHH signaling pathway (Garcia et al. 2016). The aim of this study was to assess the expression profile of 106 genes and 84 miRNAs related to SHH signaling and correlated pathways in MM and ULM. DNA methylation profiles of the promoter regions of nine SHH pathway genes were also analyzed. All genes involved directly or indirectly in the SHH pathway were included, in addition to other genes described in cancer development.

## Casuistic and methods

### Clinical sample collection

A total of 100 samples (20 MM and 80 ULM) were obtained from the Gynecology Department of the Faculdade de Medicina da Universidade de São Paulo, Brazil. Patients underwent hysterectomy or myomectomy for leiomyoma treatment between March and October 2012. All tissues were divided into matched segments derived from the same surgical specimens, with one portion frozen in liquid nitrogen and the other fixed in formalin and paraffin-embedded (FFPE). Informed consent was obtained from all participants. Clinical characteristics of the study cohort were previously reported (Garcia et al. 2016). This study was approved by the institutional review board (Protocol number 0845/11).

### miRNA microarray analysis

Twelve MM and 25 ULM FFPE samples were used for total RNA extraction using the ReliaPrep™ FFPE Total RNA Miniprep System (Promega, USA). cDNA synthesis and relative miRNA quantification were performed using the Qiagen miScript II RT Kit, Qiagen miScript SYBR Green PCR Kit, and Qiagen miScript miRNA PCR Arrays. The Breast Cancer miRNA PCR Array (Qiagen, Germany) was used to identify specific miRNA sequences. The 96-well plate was organized as follows: 84 miRNA target primers, 2 miRNA extraction controls, 6 housekeeping snRNAs (SNORD-61, SNORD-68, SNORD-72, SNORD-95, SNORD-96 A, and RNU6-2), 2 reverse transcription reaction controls, and 2 PCR positive controls. This setup enabled the assessment of 84 miRNAs potentially related to disease stage and prognosis, as well as the regulation of genes associated with cancer (Qiagen MIHS109Z). PCR was conducted on an ABI 7500 Real-Time PCR System, and relative quantification was performed using the ΔΔCt method, with the included controls verifying reverse transcription and PCR fidelity (Calvano et al. 2014).

### RT-qPCR

Total RNA from frozen tissues (20 MM and 80 ULM samples) was obtained using Trizol^®^ Reagent (Life Technologies, USA). cDNA synthesis was performed using the High-Capacity cDNA Reverse Transcription Kit (Life Technologies, USA), following the manufacturer’s instructions, with 2 µg of total RNA. Efficiency was verified by conventional PCR using primers for the β-actin gene. Relative expression of 106 genes related or associated to the SHH signaling pathway was assessed (Supplementary Table 1.). qRT-PCR reactions were conducted in duplicate with 1.2 µL of cDNA and 3.8 µL of TaqMan Gene Expression Master Mix (Applied Biosystems, USA) on a QuantStudio™ 12 K Flex OpenArray Real-Time PCR System (Life Technologies, USA), using cycling conditions recommended by the manufacturer. The housekeeping genes included *ACTB*,* B2M*,* GAPDH*,* GUSB*,* HPRT1*, and *RPLP0*. Data were analyzed using Expression Suite Software v1.0.3, employing the comparative Ct (ΔΔCt) method, using MM samples as references.

### DNA methylation analysis

Genomic DNA was extracted from 42 frozen samples (21 MM and 21 ULM) using Trizol^®^ Reagent. DNA methylation in promoter regions of *SHH*,* PTCH1*,* SMO*,* SUFU*,* GLI1*,* GLI3*,* BMP4*,* GREM1*, and *WNT1* was analyzed using Methyl-Profiler DNA Methylation qPCR Assays (SABiosciences, Germany), according to the manufacturer’s instructions. This method uses methylation-sensitive and methylation-dependent restriction enzymes to digest unmethylated and methylated DNA, respectively. The remaining DNA was quantified by real-time PCR using primers flanking the region upstream of the transcription start site. Relative concentrations of hypermethylated, unmethylated, and intermediately methylated DNA were determined by comparison with a mock digest, and data for each sample are expressed as the percentage of methylated DNA (Jaspers at al. 2010).

### Statistical analysis

Statistical analyses were performed using SPSS Statistics (version 13) and Stata MP (version 14). Data distribution was assessed using the Kolmogorov–Smirnov test. Stepwise linear regression was used to investigate the relationships among the expression levels of key SHH pathway genes. Mann–Whitney test was used to compare methylation profiles between MM and ULM samples. Fisher’s exact test was applied for categorical variables. Target genes of the studied miRNAs were identified using miRTarBase (http://mirtarbase.mbc.nctu.edu.tw/).

Correlations between selected miRNAs and their target genes were evaluated using Pearson or Spearman tests, depending on data normality. Statistical significance was set at *p* < 0.05.

## Results

**Fig. 2:**
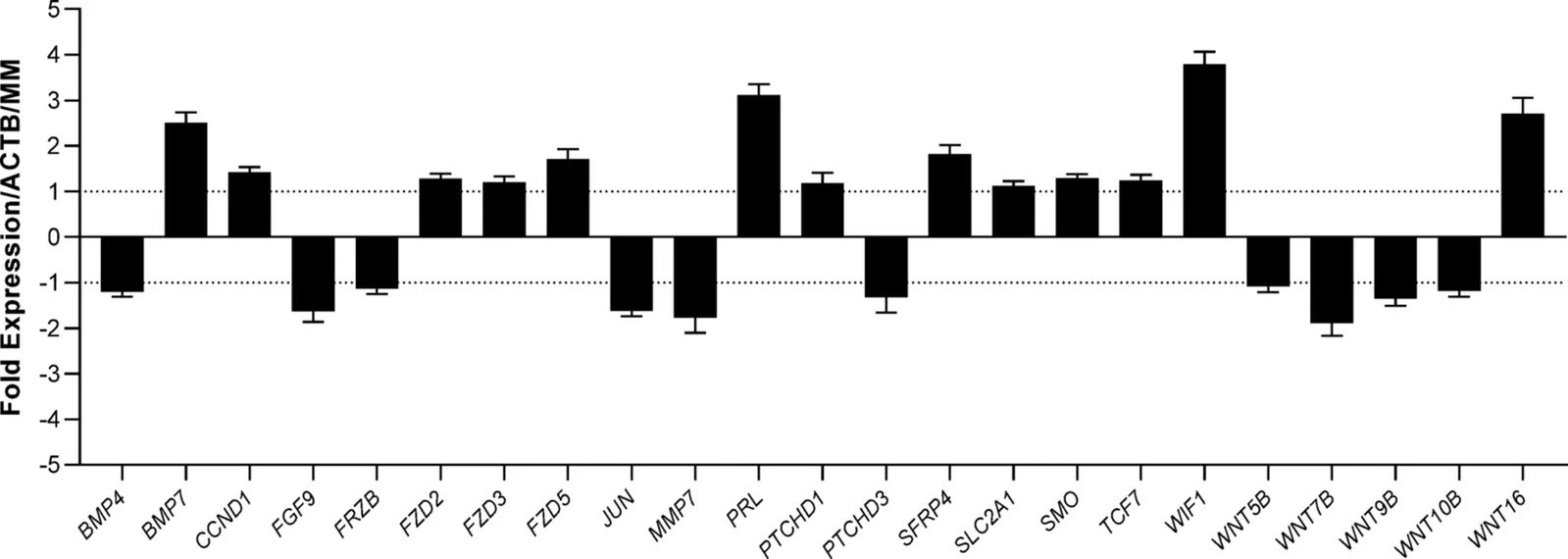
Expression profile of twenty-three genes assessed by qRT-PCR. Horizontal dashed lines represent the cutoff values determined for upregulation (fold change > 1) and downregulation (fold change < − 1) of the genes. Thirteen genes showed overexpression, while ten genes showed downregulation in ULM samples compared to the reference group (MM)

One hundred and six genes related to the Sonic Hedgehog and Wnt signaling pathways were evaluated (Supplementary Table 1.). Twenty-three genes were differentially expressed in ULM compared to MM samples (Fig. 2); 13 showed upregulation (fold change > 1) in ULM (*BMP7*,* CCND1*,* FZD2*,* FZD3*,* FZD5*,* PRL*,* PTCHD1*,* SFRP4*,* SLC2A1*,* SMO*,* TCF7*,* WIF1*, and *WNT16*), while another 10 genes were downregulated (fold change < − 1) in these samples (*BMP4*,* FGF9*,* FRZB*,* JUN*,* MMP7*,* PTCHD3*,* WNT5B*,* WNT7B*,* WNT9B*, and *WNT10B*).

**Fig. 3:**
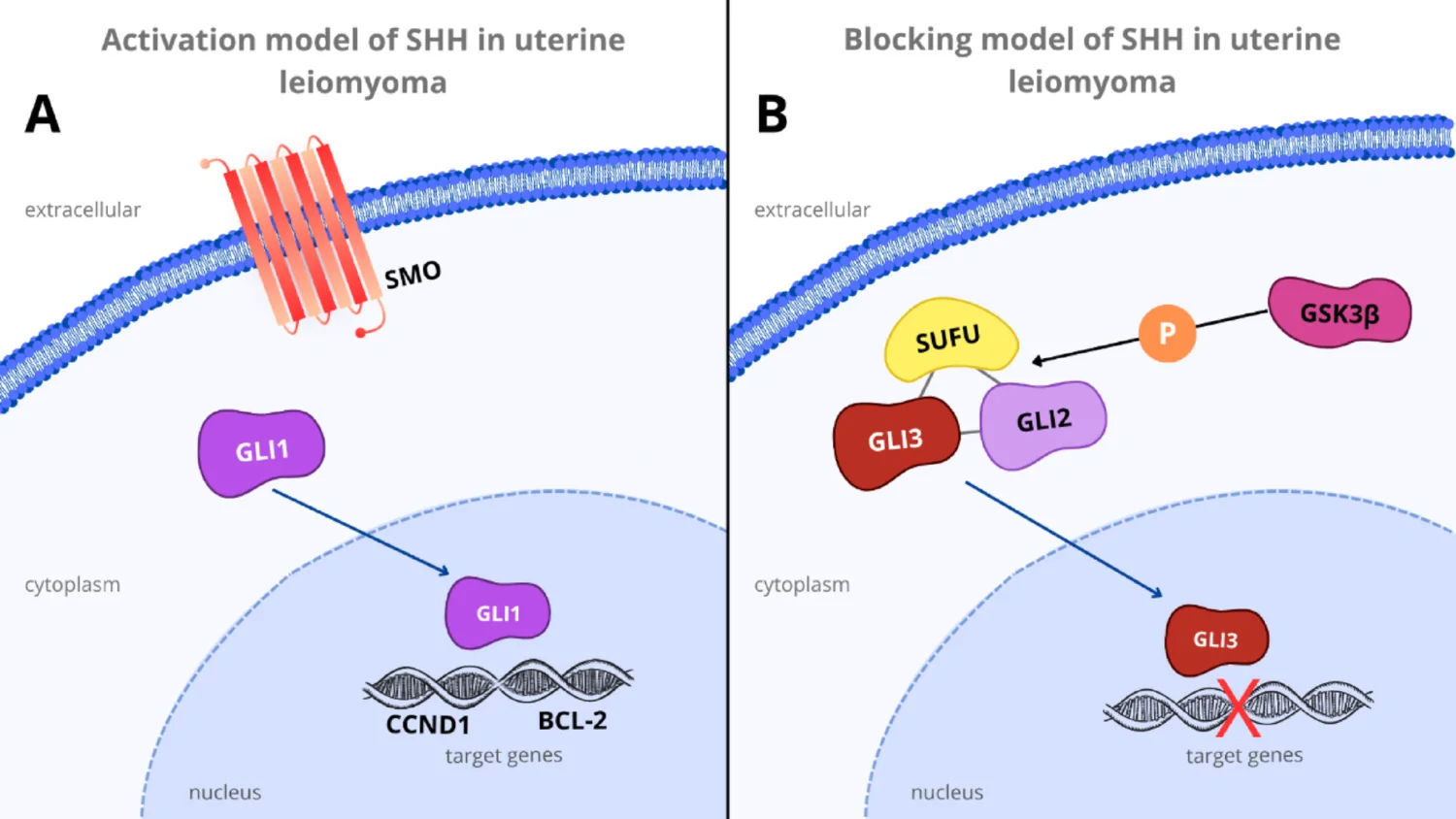
Hypothesis for Sonic Hedgehog pathway regulation in ULM based on multivariate data analysis. **A** Pathway activation mediated by increased expression of *SMO*, *GLI1*, and their target genes (*CCND1* and *BCL-2*). **B** Pathway blockade mediated by increased expression of *SUFU*, *GLI2*, *GLI3*, *GSK3β*, and inhibition of *CCND1* and *BCL*-*2*

Later, a multivariate analysis was performed to understand the activation and blocking interaction mechanisms of the SHH pathway. The main genes of the SHH signaling pathway (*SHH*,* PTCH1*,* SMO*,* SUFU*,* GLI1*,* GLI2*, and *GLI3*) and their targets (BCL-2, CCND1, and BMP4) were added one at a time. The results indicate that the combination of expression values of *GLI1*,* CCND1*, and *BCL-2* explains 50% (*p* < 0.0001) (Table 1.) of the enhanced expression previously found and described for SMO (Garcia et al. 2016). This suggests that SHH pathway activation in ULM samples is not mediated by SHH binding to the PTCH1 receptor (Fig. 3A).

Another multivariate analysis was performed using *GLI1*,* GLI2*,* GLI3*,* GSK3β*, and *SUFU* to better understand the role of *GLI1* in this tumor. The combination of *GLI2*,* GLI3*, and *GSK3β* could explain 69% (*p* < 0.0001) (Table 1.) of the higher expression levels previously described for SUFU in tumor samples compared to MM.

 0.027

**Table 1:** Stepwise regression of major genes of SHH pathway in ULM samples.

GENE	Estimate*	Std error	p-value	R² ^**^
SMO				0.50
GLI1	0.304	0.059	**0.001**	
CCND1	0.297	0.068	**0.001**	
BCL-2	0.107	0.044	**0.019**	
SUFU				0.69
GLI2	0.304	0.065	**0.001**	
GLI3	0.161	0.108	**0.001**	
GSK3β	0.437	0.070	**0.027**	

* Estimate is the standardizer regression coefficient, which allows evaluation of the relative significance of each independent variable in multiple linear regression analyses

**R² indicates the percentage of the variance explained by the independent variables in the different models

It is well documented that several patients´ clinical features (e.g., tobacco use, age at menarche, ethnicity, body mass index, number of pregnancies, and oral contraceptive use) may influence gene regulation and tumor development. In our exploratory analysis, several genes exhibited differential expression patterns when analyzed in association with specific clinical features from patients’ data in MM and ULM samples, as summarized in Table 2.. All the genes highlighted in green were downregulated, whereas genes highlighted in red were overexpressed in the tumor samples when compared to normal MM.

**Fig. 4:**
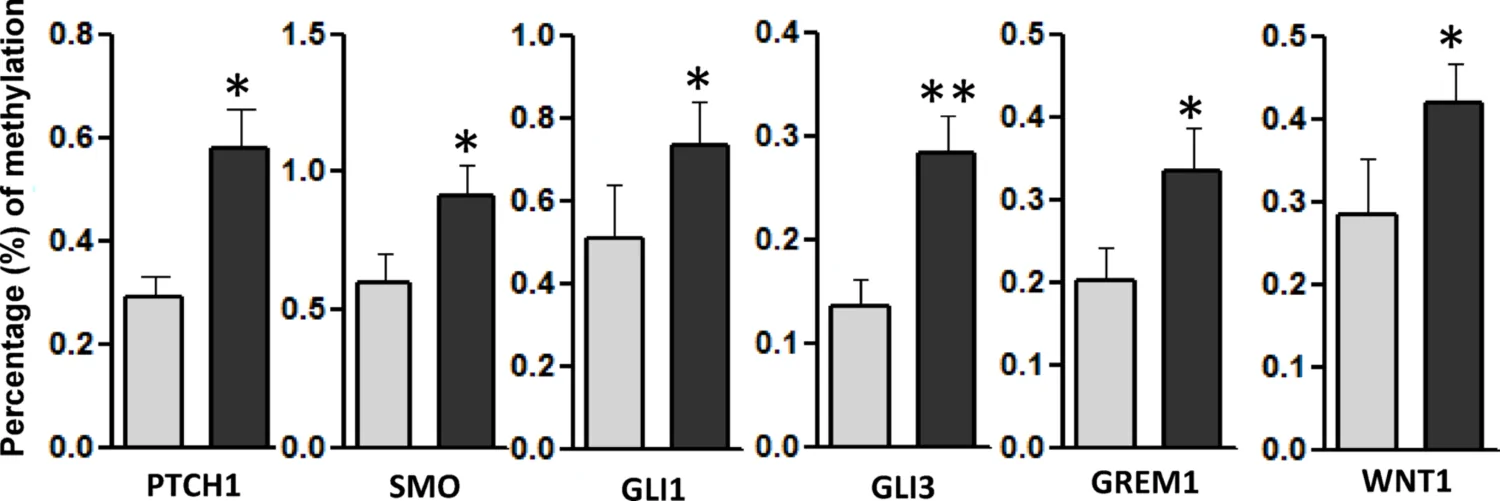
Methylation profile observed in the promoter region of the genes. The percentage of DNA methylation was significant to *PTCH1*, *SMO*, *GLI1*, *GLI3*, *GREM1*, and *WNT1* comparing the myometrium and leiomyoma. Asterisks indicate statistical significance (*p<0.05; **p<0.001)

The methylation profile of nine genes related to the SHH signaling pathway was evaluated. *GLI2* was not included due to the unavailability of a specific assay. *PTCH1*,* SMO*,* GLI1*,* GLI3*,* GREM1*, and *WNT1* genes showed significant differences between ULM and MM samples (*p* < 0.05) (Fig. 4). *SHH*,* SUFU*, and *BMP4* showed no differences. There were no associations between gene methylation levels and gene expression profiles.

**Fig. 5:**
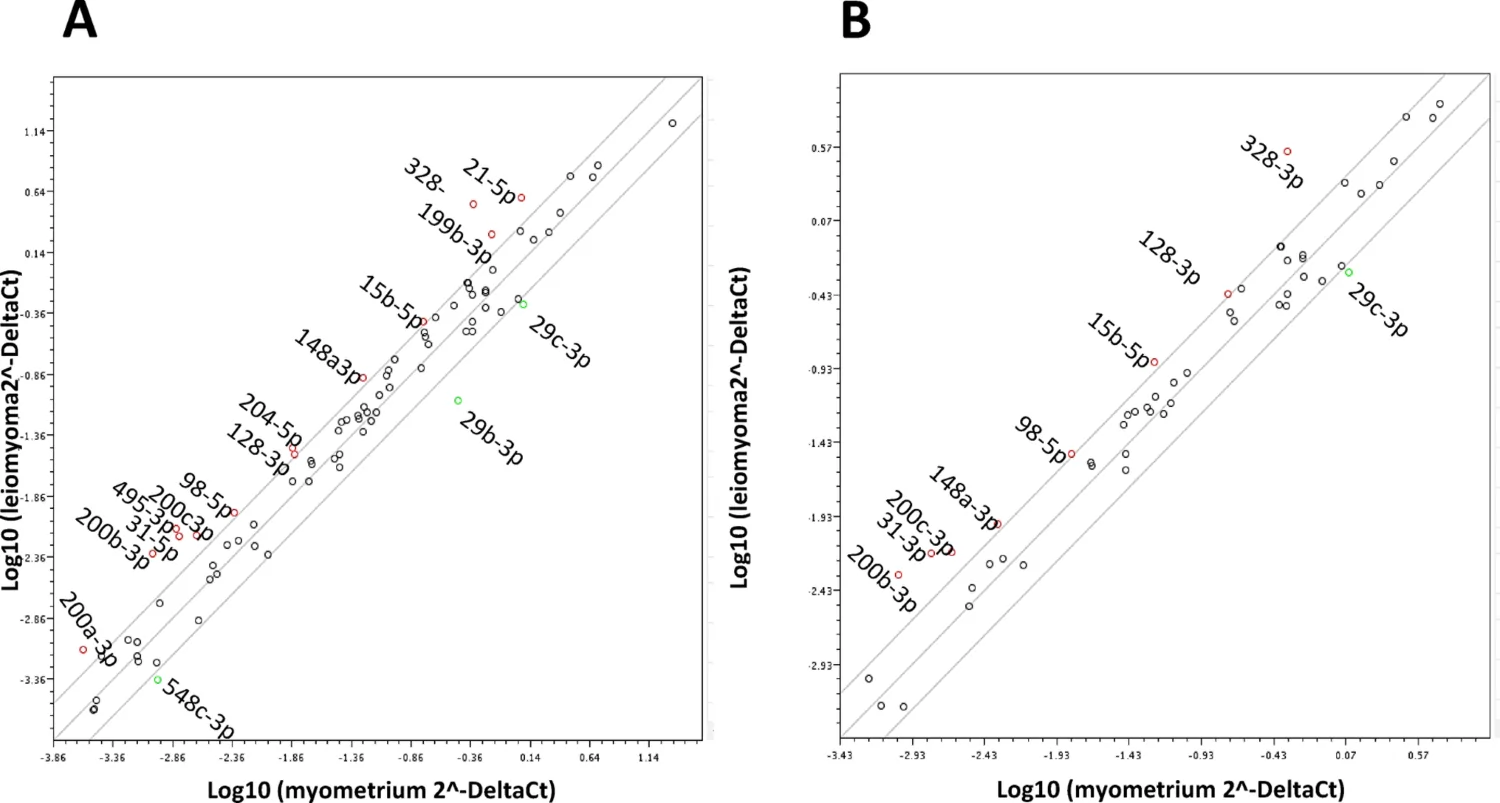
miRNA expression profile analysis comparing MM and LM samples. The graphs show miRNAs that presented overexpression (red circles, 13) and downregulation (green circles, three) (≥ 2 and ≤ − 2, respectively) and significant when *p* < 0.05. **A** Analysis of 84 miRNAs involved in cancer origin and development. **B** Out of the 84 miRNAs, 52 were identified as being related to target genes. Eight were overexpressed and one was downregulated. Graphics were created using Web-Based Data Analysis at http://www.sabiosciences.com/pcr/arrayanalysis.php

The next analysis included a total of 84 miRNAs related to cancer origin and development. They were evaluated in 12 MM and 25 ULM samples. Thirteen miRNAs were overexpressed (fold change > 2, *p* < 0.05), and three miRNAs were downregulated (fold change < − 2, *p* < 0.05) in ULM samples (Fig. 5A). Of these 84 miRNAs, 52 were identified as being related to target genes that previously showed differential expression between ULM and MM samples (Table 3.). Among these 52 miRNAs, eight were overexpressed and one was downregulated in leiomyoma samples (Fig. 5B). Next, correlations between these nine miRNAs and their target genes were analyzed. *miR-193b-3p*,* miR-19a-3p*,* miR-1-3p*,* miR-20b-5p*, and *miR-18a-5p* were positively correlated with *CCND1* expression, while *miR-206* showed a negative correlation. *miR-181a-5p* was negatively correlated with WIF1 gene expression (Table 4).

**Table 2:**
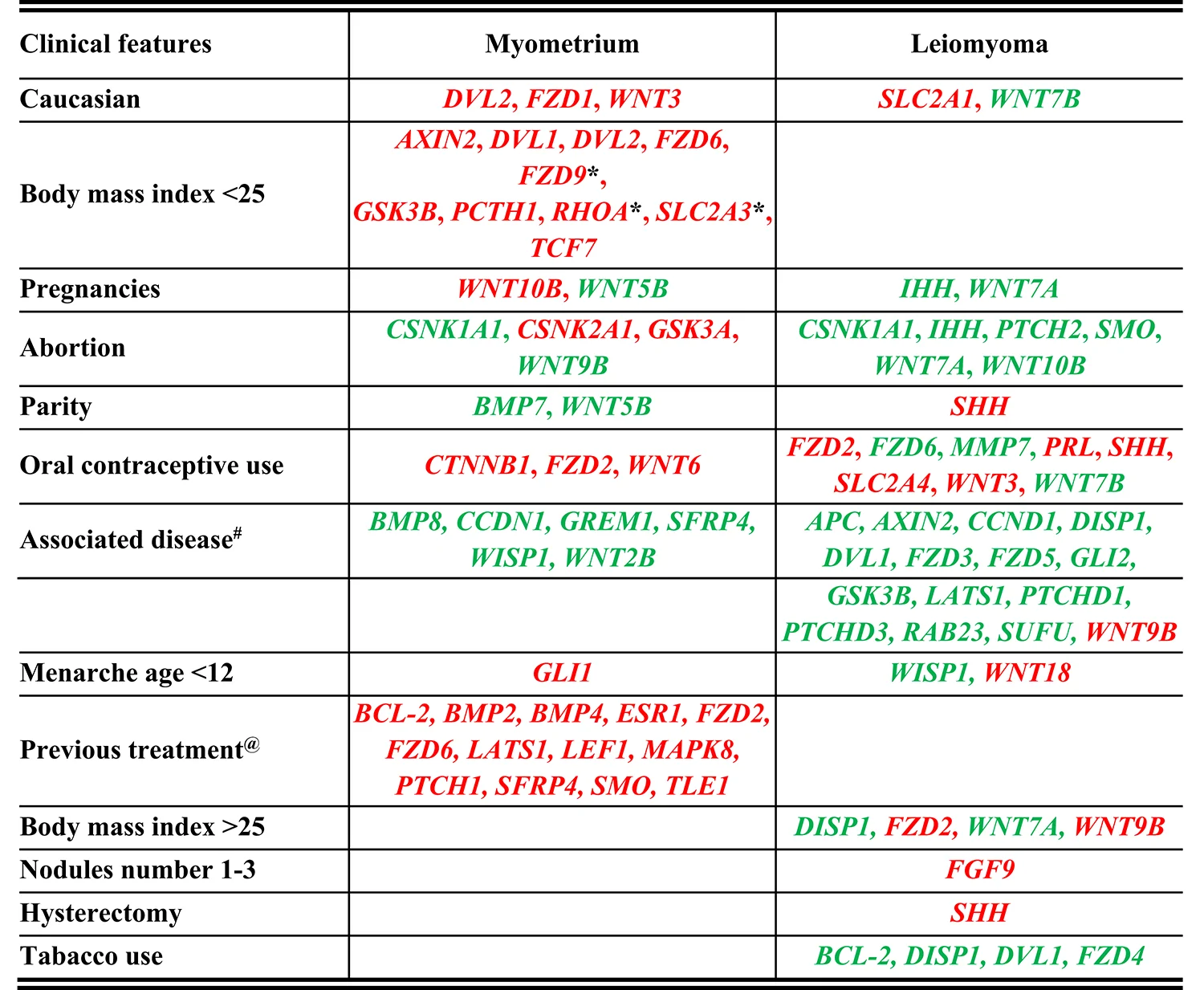
Differential gene expression profile based on the patient’s clinical features.

^*^ p-value ≤ 0.001. Green color indicates down expression of the genes, while red color indicates over expression. Fisher’s exact test was used to analyze differences among groups for categorical variables^#^ Associated diseases (pre-existing diseases): Type 2 diabetes 2, hypertension and chronic clinical conditions described by the patients^@^Previous treatment, patients who received pharmacological or hormonal therapy for LM symptoms (mainly contraceptive and GnRH analogues)

**Table 3:**
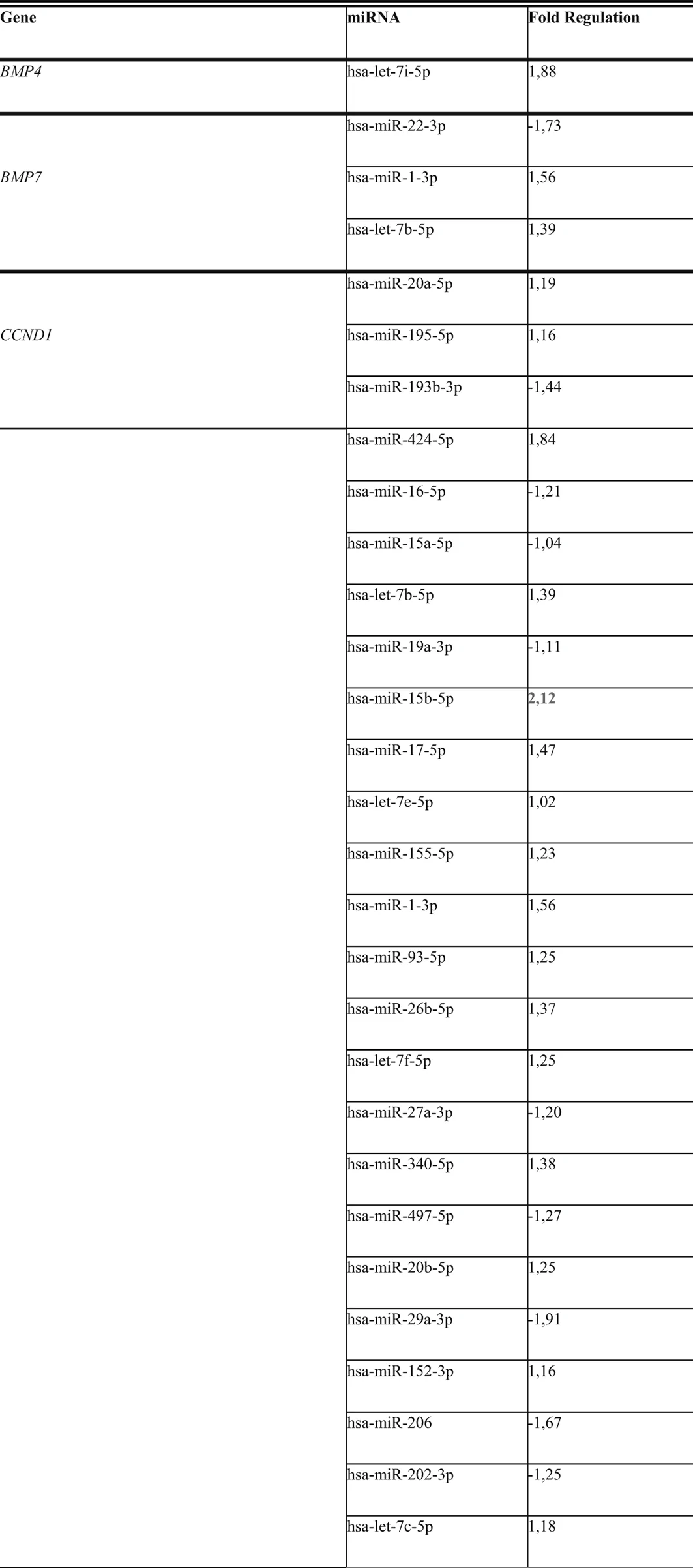

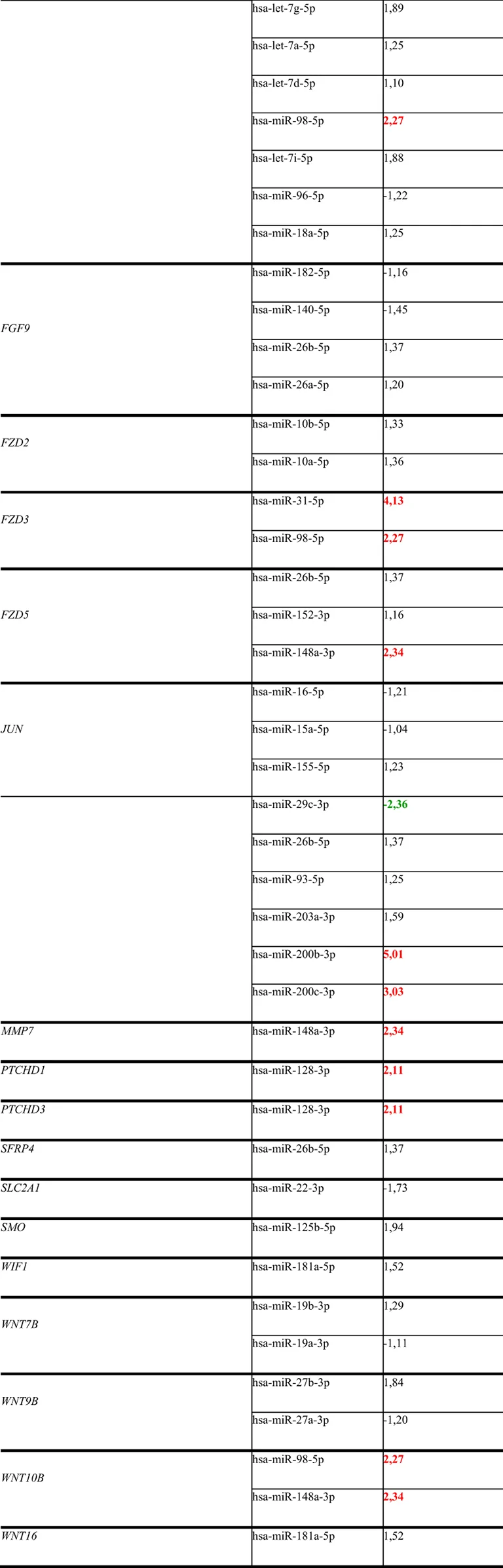
Association among 52 miRNAs with their target gene using http:/mirtarbase.mbc.natu.edu.tw/

Green and red colors indicate downregulation and upregulation, respectively. All miRNA target genes listed herein are included among the 106 SHH related genes previously analyzed by qRT-PCR in ULM and MM samples

**Table 4:** Single correlation between miRNAs and target genes

Signaling Pathway	Gene	miRNA	*r* *	*p* value**
*SHH*	*CCND1*	miR-193b-3p	0.63	**0.002**
		miR-206*	−0.47	**0.03**
		miR-19a-3p	0.60	**0.003**
		miR-17-5p*	0.40	0.07
		miR-1-3p*	0.60	**0.003**
		miR-20b-5p*	0.53	**0.01**
		miR-18a-5p	0.56	**0.01**
	*PTCHD1*	miR-128-3p*	0.34	0.13
*Wnt*	*WIF1*	miR-181a-5p*	−0.53	**0.01**

*r- correlation coefficient

**Non-parametric values were evaluated by Spearman’s correlation

## Discussion

Research on ULM has revealed that genetic mutations, dysregulated signaling pathways, and epigenetic mechanisms are significantly involved in their development (dos Anjos et al. 2019; Carbajo-García et al. 2021). Additionally, previous studies have identified pathways such as SHH and Wnt that participate in extracellular matrix accumulation, cell proliferation, and tumor growth (Garcia et al. 2021b; El Sayed et al. 2025). Epigenetic modifications, including changes in DNA methylation, and miRNA regulation have also emerged as important layers of regulation that influence gene expression in these tumors (Baranov et al. 2019; Carbajo-García et al. 2021). However, the integrated crosstalk between these regulatory levels remains to be fully elucidated, which was the focus of our investigation.

Significant changes in gene expression were observed between ULM and MM tissues, allowing the identification of relevant molecular patterns involved in their differentiation process. To further contextualize our findings, a comparative overview of the 23 differentially expressed genes in our study was conducted with transcriptomic datasets for ULM versus MM. This evaluation revealed that a notable proportion of the profile observed in our cohort is also exhibited in the literature, thereby validating the findings and underscoring the robustness of the observed differences (Mehine et al. 2016; Berta et al. 2021). In the dataset analyzed by Mehine et al. (2016), there was a consistency in the gene expression pattern for the majority of the genes evaluated, with *TCF7* being the main exception, as it was found to be downregulated in that study and upregulated in our analysis. In the study conducted by Berta et al. (2021), the pattern was more variable. Some genes showed a similar pattern to our cohort, while *BMP4*, *FGF9*, *FRZB*, *JUN*, *MMP7*, *PTCHD3*, *WNT5B*, *WNT9B*, and *WNT10B* exhibited an opposite behaviour.

Expression levels of *SMO* and *PTCHD1* were found to be increased, while *PTCHD3* expression was reduced. These findings suggest a potential loss of inhibitory control and, consequently, activation of the SHH pathway. This interpretation is supported by findings in other tumors, in which dysregulation of SHH components has been associated with enhanced proliferation and altered differentiation (Kotulak-Chrzaszcz et al. 2022). The upregulation of *CCND1*, a fundamental G1/S cell cycle regulator and downstream target of SHH via GLI transcription factors, reinforces pathway activation (Kotulak-Chrzaszcz et al. 2022). Additionally, the observed increase in *BMP7* suggests a combined effect with SHH signaling, as *BMP7* has been shown to enhance SHH and GLI1 activity in other cell types, promoting downstream gene activation and proliferation (Feltran et al. 2024). These findings collectively suggest that altered regulation of SHH components, in combination with elevated *BMP7* and *CCND1* expression, may play a role in promoting tumor growth and altered cell differentiation in ULMs.

As part of the complex signaling network in ULM biology, the SHH pathway may act alongside Wnt signaling. The increased expression of Frizzled family genes (*FZD2*,* FZD3*, and *FZD5*) and *TCF7*, alongside the downregulation of *FRZB* and the upregulation of the inhibitors *SFRP4* and *WIF1*, suggests that the Wnt signaling pathway remains active, even though its inhibitors are overexpressed in ULM tissues. In addition, changes in Wnt ligands were also observed: while *WNT5B*,* WNT7B*,* WNT9B*, and *WNT10B* were downregulated, *WNT16* was upregulated. This finding contrasts with Mangioni et al. (2005), who reported *WNT5B* overexpression in ULMs. This suggests that Wnt ligand regulation may vary depending on tumor context or experimental conditions. Our pattern may reflect a selective and context-dependent modulation of Wnt signaling. These findings are consistent with previous studies, such as Harada et al. (2021), which showed that FZD5 helps prevent cellular senescence by maintaining cells in a proliferative state. A similar mechanism may be occurring in ULM, in which changes in Wnt signaling support tumor growth and inhibit normal cellular differentiation (Harada et al. 2021). This profile suggests a possible partial activation of the canonical pathway associated with increased cell proliferation, while the upregulation of WNT16 may suggest the involvement of the non-canonical pathway (Williamson et al. 2025). Additionally, Ono et al. (2013) demonstrated that estrogen and progesterone can induce *WNT11* and *WNT16* expression in mature ULM cells, promoting tumor growth and supporting the relevance of these ligands in ULM biology. Due to the complexity of this system, in which receptors, ligands, and inhibitors interact in an intricate manner, further research is required to better understand the role of each component in both canonical and non-canonical processes (El Sabeh et al. 2021).

Moreover, SHH pathway activation in leiomyomas may not depend solely on the classic SHH - PTCH1 interaction. Multivariate analysis showed that approximately 50% of SMO expression could be explained by the combined expression of *GLI1*,* CCND1*, and *BCL-2*, suggesting alternative activation routes. This pattern is similar to what has been observed in gastrointestinal stromal tumors (GISTs), in which high GLI1 expression is maintained even when SHH levels are low (Yoshizaki et al. 2006). Although ULM and GISTs are distinct entities, both share mesenchymal characteristics that may support similar regulatory mechanisms involving these proteins. In addition, the presence of increased GLI1 levels in low-SHH tumors supports the contribution of other pathways, such as GSK3β and other GLI transcription factors (Yoshizaki et al. 2006). Indeed, *GLI2*,* GLI3*, and *GSK3β* together explained 69% of the variation in SUFU expression, reinforcing the concept of multifactorial regulation. These findings suggest more complex SHH signaling dynamics in leiomyomas, which may help guide targeted therapies toward alternative mechanisms of pathway regulation.

The methylation profile of the promoter regions of *PTCH1*,* SMO*,* GLI1*, *GLI3*,* GREM1*, and *WNT1* demonstrated significant differences when comparing ULM and MM samples, suggesting potential epigenetic modulation of the SHH pathway in ULM. *SHH*,* SUFU*, and *BMP4* showed no changes, and no correlation was found between methylation and gene expression profiles. This absence of correlation may suggest that gene regulation in ULM may involve methylation outside core promoter regions, distal enhancer activity, and/or modulation by miRNAs and transcription factors.

Additionally, the lack of prior data regarding this interaction emphasizes the importance of future experimental studies to elucidate the underlying molecular mechanisms. Previous studies have demonstrated that *PTCH1* promoter hypermethylation occurs in various tumors, including dermoid cysts, ovarian fibromas, breast cancer, rhabdomyosarcoma, medulloblastomas, and astrocytomas, potentially reducing its inhibitory effect on the SHH pathway and contributing to altered signaling (Garcia et al. 2021a; Skoda et al. 2018). These observations are consistent with the hypermethylation patterns identified in the present study. Methylation patterns have been shown to reveal specific signatures that may be useful as diagnostic and prognostic biomarkers, assisting in distinguishing between ULM subtypes and other rare uterine smooth muscle tumors (Mlodawska et al. 2022). These data underscore the significance of epigenetic approaches in enhancing the diagnosis, prognosis, and classification of these lesions.

Several studies have demonstrated that certain miRNAs display significantly altered expression patterns in ULM compared with MM. These findings suggest a direct relationship with the etiology of these lesions and reinforce the involvement of these pathways in ULM tumorigenesis (Kim et al. 2022). Our analyses revealed potential post-transcriptional regulatory mechanisms associated with differentially expressed genes. In the case of *CCND1*, which was overexpressed, we identified two miRNAs that were also increased in ULM (*miR-15b-5p* and *miR-98-5p*), as well as five others (*miR-193b-3p*,* miR-19a-3p*,* miR-1-3p*,* miR-20b-5p*, and *miR-18a-5p*) that were positively correlated with its expression. Although miRNAs typically function as repressors of their targets (Marsh et al. 2008), the positive correlations observed suggest coordinated regulation or escape from post-transcriptional control, underscoring the critical role of CCND1 in cell proliferation. Despite being described as a tumor suppressor by the Wnt/β-catenin pathway in hepatocellular carcinoma by Zhang et al. (2017), *miR-98-5p* was increased simultaneously with *CCND1*, indicating that this miRNA alone does not control *CCND1* expression in ULM. In contrast, *miR-206* showed a negative correlation with *CCND1*, suggesting a repressive role in this context. Supporting this interpretation, studies in ovarian cancer have shown that *miR-206* reduces CCND1 and CCND2 expression, reinforcing its role as a negative regulator (Chang et al. 2018).

In the case of *JUN*, whose expression was reduced, its inhibitors *miR-200b-3p* and *miR-200c-3p* were overexpressed (Ciebiera et al. 2020). Their presence may partially explain the observed decrease in JUN, which plays a role in extracellular matrix regulation. Furthermore, a decrease in *miR-29c-3p*, which also regulates *JUN*, was observed. Theoretically, this reduction would favor increased JUN expression and could contribute to the fibrotic phenotype of ULM .(Anjos, et al., 2019); Marsh et al. 2016); however, it appears insufficient to counteract *JUN* downregulation in these samples.

The expression levels of *FZD3* and *FZD5*, along with their regulatory *miR-31-5p* and *miR-98-5p*, and *miR-148a-3p*, respectively, were increased in ULM. This upregulation suggests that the expression of these genes may bypass post-transcriptional repression typically mediated by these miRNAs. As both genes encode receptors involved in Wnt signaling, their sustained expression supports the hypothesis that this pathway is active in ULM tissue. Studies have demonstrated that *miR-98-5p* functions as a negative regulator of the Wnt pathway in several cancer types, including ovarian cancer, glioblastoma, and colorectal cancer, by inhibiting receptor expression such as FZD3 (Kenneth et al. 2023). However, although *miR-31-5p* is predicted to target *FZD3*, it has also been associated with oncogenic activity in certain tumor contexts (Kenneth et al. 2023). This raises the possibility that, in ULM, its increased expression may contribute to disease progression rather than pathway suppression. Taken together, the simultaneous presence of *FZD3* and these *miRNAs* may reflect a regulatory imbalance, with distinct or opposing roles, in which *miR-31-5p* potentially promotes Wnt pathway activation while the inhibitory effect of *miR-98-5p* may be insufficient to counteract it. Similarly, although *WNT10B* expression was reduced, this change was accompanied by increased levels of *miR-98-5p* and *miR-148a-3p*, suggesting that these miRNAs may directly contribute to its repression. While the role of *miR-148a-3p* as a regulator of the Wnt/β-catenin pathway has been described in liver cell models (Hu et al. 2018), its modulation may also be relevant in ULM, in which Wnt signaling plays a crucial role in cell proliferation and pathogenesis.

*WIF1* showed a negative correlation with *miR-181a-5p*. Previous studies have described the inhibitory action of *miR-181a-5p* on *WIF1* in other clinical contexts, such as hepatopulmonary syndrome (Li et al. 2023). Our results suggest that this miRNA may be involved in modulating or limiting *WIF1* overexpression, possibly as part of a compensatory mechanism in ULM.

Regarding *MMP7*, which was reduced in ULM, increased levels of *miR-148a-3p* were identified, suggesting functional downregulation. Sakamoto et al. (2014), studying gastric cancer, confirmed that increased *miR-148a-3p* levels suppress MMP7 expression, implying a negative regulatory relationship between these molecules (Sakamoto et al. 2014). *PTCHD1* and *PTCHD3*, both members of the Hedgehog pathway, exhibited distinct expression patterns: *PTCHD1* was upregulated, whereas *PTCHD3* was downregulated, despite both being associated with *miR-128-3p*, which was also increased. Interestingly, only *PTCHD3* showed a negative functional correlation, suggesting selective gene-specific regulation. As no previous studies have described this relationship, further investigation is required to better understand this mechanism.

These findings support a model in which SHH signaling is predominantly activated through non-canonical mechanisms, while Wnt signaling remains partially active despite the concomitant overexpression of inhibitory components. In this context, miRNAs appear to modulate, rather than fully suppress, key regulatory nodes within both pathways, contributing to a permissive molecular environment that sustains cell proliferation in ULM. Overall, these results highlight the complex interplay between signaling pathways, epigenetic regulation, and post-transcriptional control in uterine leiomyoma pathogenesis. Importantly, this integrative regulatory framework provides potential molecular candidates for biomarker development and therapeutic targeting. Further functional and mechanistic studies are warranted to validate these interactions and to determine their clinical relevance in ULMs.

## Conclusions

In summary, our findings demonstrate significant molecular alterations in ULM compared with MM, involving a complex regulatory network associated with the SHH and Wnt signaling pathways. Evidence of SHH pathway activation is supported by the increased expression of key components, such as *SMO* and *CCND1*, which are known to promote tumor growth and cell proliferation. Concurrently, the Wnt signaling pathway appears to remain active despite the overexpression of its canonical inhibitory components, suggesting the presence of a multifaceted regulatory mechanism that favors a proliferative cellular phenotype.

Furthermore, SHH pathway activation in ULM may occur through alternative regulatory routes, as indicated by multivariate analyses. Epigenetic alterations, particularly DNA methylation of genes such as PTCH1, may contribute to this process, highlighting the importance of epigenetic regulation in ULM pathogenesis. In addition, miRNA profiling revealed an intricate post-transcriptional regulatory landscape, in which certain miRNAs act as suppressors, whereas others appear insufficient to effectively regulate the expression of key targets, including *CCND1*.

Collectively, these findings contribute to a more comprehensive understanding of the molecular mechanisms underlying ULM development and progression. Moreover, the identified alterations in SHH and Wnt signaling pathways, together with their epigenetic and post-transcriptional regulators, may represent promising candidates for diagnostic and prognostic biomarkers, as well as potential targets for the development of more precise therapeutic strategies.

## Supplementary Information

Below is the link to the electronic supplementary material.


Supplementary Material 1

