# Supplementary material for: Molecular insights into uterine fibroids: crosstalk between SHH and Wnt pathways, DNA methylation, and miRNAs regulation
Source: Mol Genet Genomics. 2026 Aug 29;301(1):180. doi: 10.1007/s00438-026-02491-3 (PMC13526075; doi:10.1007/s00438-026-02491-3)
Supplement: Supplementary file 1 [file 438_2026_2491_MOESM1_ESM.docx]

**Supplementary Table1:** Genes evaluated in the qRT-PCR analysis

| **Symbol** | **Gene** | **Assay Identification *TaqMan*®** | **Reference** |
| --- | --- | --- | --- |
| *ACTB** | Actin, beta | hs 99999903_m1 | NM_001101 |
| *B2M** | Beta-2-microglobulin | hs 00984230_m1 | NM_004048 |
| *GAPDH** | Glyceraldehyde-3-phosphate dehydrogenase | hs 99999905_m1 | NM_002046 |
| *GUSB** | Glucuronidase, beta | hs 00939627_m1 | NM_000181 |
| *HPRT1** | Hypoxanthine phosphoribosyltransferase 1 | hs 02800695_m1 | NM_000194 |
| *RPLP0** | Ribosomal protein, large, P0 | hs 99999902_m1 | NM_001002 |
| *APC* | Adenomatous polyposis coli | hs 01568269_m1 | NM_000038 |
| *AXIN1* | Axin 1 | hs 00394718_m1 | NM_003502 |
| *AXIN2* | Axin 2 | hs 00610344_m1 | NM_004655 |
| *BCL2* | B-cell CLL/lymphoma 2 | hs 00608023_m1 | NM_000633 |
| *BMP2* | Bone morphogenetic protein 2 | hs 00154192_m1 | NM_001200 |
| *BMP4* | Bone morphogenetic protein 4 | hs 01041266_m1 | NM_130851 |
| *BMP5* | Bone morphogenetic protein 5 | hs 00234930_m1 | NM_021073 |
| *BMP6* | Bone morphogenetic protein 6 | hs 01099594_m1 | NM_001718 |
| *BMP7* | Bone morphogenetic protein 7 | hs 00233476_m1 | NM_001719 |
| *BMP8B* | Bone morphogenetic protein 8b | hs 01629120_s1 | NM_001720 |
| *CCND1* | Cyclin D1 | hs 00765553_m1 | NM_053056 |
| *CCND2* | Cyclin D2 | hs 00153380_m1 | NM_001759 |
| *CSNK1A1* | Casein kinase 1, alpha 1 | hs 00793391_m1 | NM_001892 |
| *CSNK2A1* | Casein kinase 2, alpha 1 polypeptide | hs 00953536_m1 | NM_001895 |
| *CTNNB1* | Catenin (cadherin-associated protein), beta 1, 88kDa | hs 00355049_m1 | NM_001904 |
| *CTNNBIP1* | Catenin, beta interacting protein 1 | hs 00172016_m1 | NM_020248 |
| *DAAM1* | Dishevelled associated activator of morphogenesis 1 | hs 00323674_m1 | NM_014992 |
| *DHH* | Desert hedgehog | hs 00368306_m1 | NM_021044 |
| *DISP1* | Dispatched homolog 1 (Drosophila) | hs 00399179_m1 | NM_032890 |
| *DISP2* | Dispatched homolog 2 (Drosophila) | hs 00394338_m1 | NM_033510 |
| *DVL1* | Dishevelled, dsh homolog 1 (Drosophila) | hs 00182896_m1 | NM_004421 |
| *DVL2* | Dishevelled, dsh homolog 2 (Drosophila) | hs 00182901_m1 | NM_004422 |
| *EIF5A* | eucariotic initiation factor 5A | hs 04188519_m1 | NM_001143 |
| *ERBB4* | V-erb-a erythroblastic leukemia viral oncogene homolog 4 (avian) | hs 00955525_m1 | NM_005235 |
| *ESR1* | Estrogen receptor | hs 00174860_m1 | NM_001253 |
| *FGF4* | Fibroblast growth factor 4 | hs 00999691_m1 | NM_002007 |
| *FGF9* | Fibroblast growth factor 9 (GLIa-activating factor) | hs 00181829_m1 | NM_002010 |
| *FGFR3* | Fibroblast growth factor receptor 3 | hs 00179829_m1 | NM_000142 |
| *FOXE1* | Forkhead box E1 (thyroid transcription factor 2) | hs 00916085_s1 | NM_004473 |
| *FOXN1* | Forkhead box N1 | hs 00186096_m1 | NM_003593 |
| *FOXO3A* | Forkhead box | hs 00818121_m1 | NM_001455 |
| *FRZB* | Frizzled-related protein | hs 00173503_m1 | NM_001463 |
| *FZD1* | Frizzled family receptor 1 | hs 00268943_s1 | NM_003505 |
| *FZD2* | Frizzled family receptor 2 | hs 00361432_s1 | NM_001466 |
| *FZD3* | Frizzled family receptor 3 | hs 00184043_m1 | NM_017412 |
| *FZD4* | Frizzled family receptor 4 | hs 00201853_m1 | NM_012193 |
| *FZD5* | Frizzled family receptor 5 | hs 00258278_s1 | NM_003468 |
| *FZD6* | Frizzled family receptor 6 | hs 00171574_m1 | NM_003506 |
| *FZD7* | Frizzled family receptor 7 | hs 00275833_s1 | NM_003507 |
| *FZD8* | Frizzled family receptor 8 | hs 00259040_s1 | NM_031866 |
| *FZD9* | Frizzled family receptor 9 | hs 00268952_s1 | NM_003508 |
| *GAS1* | Growth arrest-specific 1 | hs 00266715_s1 | NM_002048 |
| *GLI1* | GLI family zinc finger 1 | hs 01110766_m1 | NM_005269 |
| *GLI2* | GLI family zinc finger 2 | hs 01119974_m1 | NM_005270 |
| *GLI3* | GLI family zinc finger 3 | hs 00609233_m1 | NM_000168 |
| *GREM1* | Gremlin 1 | hs 00171951_m1 | NM_013372 |
| *GSK3A* | Glycogen synthase kinase 3 alpha | hs 00997938_m1 | NM_019884 |
| *GSK3B* | Glycogen synthase kinase 3 beta | hs 01047719_m1 | NM_002093 |
| *HHAT* | Hedgehog acyltransferase | hs 00911326_m1 | NM_018194 |
| *HHIP* | Hedgehog interacting protein | hs 01011015_m1 | NM_022475 |
| *IFT52* | Intraflagellar transport 52 homolog (Chlamydomonas) | hs 00211198_m1 | NM_016004 |
| *IHH* | Indian hedgehog | hs 01081801_m1 | NM_002181 |
| *JUN* | Jun proto-oncogene | hs 01103582_s1 | NM_002228 |
| *LATS1* | LATS, large tumor suppressor, homolog 1 (Drosophila) | hs 01125528_m1 | NM_004690 |
| *LATS2* | LATS, large tumor suppressor, homolog 2 (Drosophila) | hs 00324396_m1 | NM_014572 |
| *LEF1* | Lymphoid enhancer-binding factor 1 | hs 01547250_m1 | NM_016269 |
| *MAPK1* | Mitogen-activated protein kinase 1 | hs 01046830_m1 | NM_002745 |
| *MMP7* | Matrix metallopeptidase 7 (matrilysin, uterine) | hs 01042796_m1 | NM_002423 |
| *MAPK8* | Mitogen-activated protein kinase 8 | hs 00177083_m1 | NM_002750 |
| *MTSS1* | Metastasis suppressor 1 | hs 00207341_m1 | NM_014751 |
| *MYC* | V-myc myelocytomatosis viral oncogene homolog (avian) | hs 00153408_m1 | NM_002467 |
| *PGR* | progesterone receptor | hs 01556702_m1 | NM_000926 |
| *PRL* | prolaction | hs 00168730_m1 | NM_000948 |
| *PRLR* | prolactin receptor | hs 01061477_m1 | NM_000949 |
| *PTCH1* | Patched 1 | hs 00181117_m1 | NM_000264 |
| *PTCH2* | Patched 2 | hs 00184804_m1 | NM_003738 |
| *PTCHD1* | Patched domain containing 1 | hs 00288486_m1 | NM_173495 |
| *PTCHD2* | Patched domain containing 2 | hs 01367724_m1 | NM_020780 |
| *PTCHD3* | Patched domain containing 3 | hs 01584645_m1 | NM_001034 |
| *PTEN* | protein tensin homologue | hs 02621230_s1 | NM_000314 |
| *RAB23* | RAB23, member RAS oncogene family | hs 00212407_m1 | NM_183227 |
| *RHOA* | Ras homolog gene family, member A | hs 00357608_m1 | NM_001664 |
| *SFRP1* | Secreted frizzled-related protein 1 | hs 00610060_m1 | NM_003012 |
| *SFRP4* | Secreted frizzled-related protein 4 | hs 00180066_m1 | NM_003014 |
| *SHH* | Sonic hedgehog | hs 00179843_m1 | NM_000193 |
| *SLC2A1* | Facilitated glucose transporter member 1 | hs 00892681_m1 | NM_001455 |
| *SLC2A3* | Facilitated glucose transporter member 3 | hs 00359840_m1 | NM_006931 |
| *SLC2A4* | Facilitated glucose transporter member 4 | hs 00168966_m1 | NM_001042 |
| *SMO* | Smoothened, frizzled family receptor | hs 01090242_m1 | NM_005631 |
| *SUFU* | Suppressor of fused homolog (Drosophila) | hs 00171981_m1 | NM_016169 |
| *TCF7* | Transcription factor 7 (T-cell specific, HMG-box) | hs 00175273_m1 | NM_003202 |
| *TCF7L1* | Transcription factor 7-like 1 (T-cell specific, HMG-box) | hs 01064103_m1 | NM_031283 |
| *TLE1* | Transducin-like enhancer of split 1 (E(sp1) homolog, Drosophila) | hs 00270768_m1 | NM_005077 |
| *TP53* | Tumor protein p53 | hs 01034249_m1 | NM_000546 |
| *VEGFA* | Vascular endothelial growth factor A | hs 00900055_m1 | NM_003376 |
| *WIF1* | WNT inhibitory factor 1 | hs 00183662_m1 | NM_007191 |
| *WISP1* | WNT1 inducible signaling pathway protein 1 | hs 04234730_m1 | NM_003882 |
| *WNT1* | Wingless-type MMTV integration site family, member 1 | hs 01011247_m1 | NM_005430 |
| *WNT10A* | Wingless-type MMTV integration site family, member 10A | hs 00228741_m1 | NM_025216 |
| *WNT10B* | Wingless-type MMTV integration site family, member 10B | hs 00559664_m1 | NM_003394 |
| *WNT11* | Wingless-type MMTV integration site family, member 11 | hs 00182986_m1 | NM_004626 |
| *WNT16* | Wingless-type MMTV integration site family, member 16 | hs 00365138_m1 | NM_057168 |
| *WNT2* | Wingless-type MMTV integration site family member 2 | hs 00608224_m1 | NM_003391 |
| *WNT2B* | Wingless-type MMTV integration site family, member 2B | hs 00921614_m1 | NM_004185 |
| *WNT3* | Wingless-type MMTV integration site family, member 3 | hs 00902257_m1 | NM_030753 |
| *WNT3A* | Wingless-type MMTV integration site family, member 3A | hs 00263977_m1 | NM_033131 |
| *WNT4* | Wingless-type MMTV integration site family, member 4 | hs 01573504_m1 | NM_030761 |
| *WNT5A* | Wingless-type MMTV integration site family, member 5A | hs 00998537_m1 | NM_003392 |
| *WNT5B* | Wingless-type MMTV integration site family, member 5B | hs 01086864_m1 | NM_032642 |
| *WNT6* | Wingless-type MMTV integration site family, member 6 | hs 00362452_m1 | NM_006522 |
| *WNT7A* | Wingless-type MMTV integration site family, member 7A | hs 01114990_m1 | NM_004625 |
| *WNT7B* | Wingless-type MMTV integration site family, member 7B | hs 00536497_m1 | NM_058238 |
| *WNT8A* | Wingless-type MMTV integration site family, member 8A | hs 00230534_m1 | NM_058244 |
| *WNT8B* | Wingless-type MMTV integration site family, member 8B | hs 00610126_m1 | NM_003393 |
| *WNT9A* | Wingless-type MMTV integration site family, member 9A | hs 00243321_m1 | NM_003395 |
| *WNT9B* | Wingless-type MMTV integration site family, member 9B | hs 00287409_m1 | NM_003396 |

**Legend:** * house keeping genes
